# The Effectiveness of Individual Mental Health Interventions for Depressive, Anxiety and Conduct Disorder Symptoms in School Environment for Adolescents Aged 12–18—A Systematic Review

**DOI:** 10.3389/fpsyt.2021.779933

**Published:** 2021-12-09

**Authors:** Johanna Karukivi, Outi Herrala, Elina Säteri, Anna Tornivuori, Sanna Salanterä, Minna Aromaa, Kim Kronström, Max Karukivi

**Affiliations:** ^1^Department of Adolescent Psychiatry, Turku University Hospital, University of Turku, Turku, Finland; ^2^Department of Nursing Science, Turku University Hospital, University of Turku, Turku, Finland; ^3^Department of Public Health, University of Turku, Turku, Finland; ^4^Outpatient Clinic of Children and Adolescents, Turku, Finland; ^5^Psychiatric Care Division, Satakunta Hospital District, Pori, Finland

**Keywords:** cognitive-behavioral therapy (CBT), intervention, mental health, school, systematic (literature) review

## Abstract

**Background:** Mental health problems are a major health issue for children and adolescents around the world. The school environment allows adolescents to be reached comprehensively and on a low threshold, making it a potential environment for mental health interventions. The aim of this review was to describe interventions delivered by health-care workers in school environment for individual adolescents aged 12–18 with mental health problems and to assess the effectiveness of these interventions.

**Methods:** This systematic review was conducted in adherence with the PRISMA guidelines. Altogether 349 studies were screened and 24 of them were included in full text assessment. Eight studies were included in the qualitative synthesis. Only in three studies the intervention was compared to another intervention or the study setting included a control group. Five of the interventions were based on cognitive-behavioral therapy and three on other approaches. In seven studies, one of the main response variables was based on assessment of depressive symptoms and/or a depressive disorder. The quality of the studies was limited with notable risk for bias for some studies.

**Results:** Based on reported symptom reductions, for most of the interventions, the results were good. Symptom reductions were also typically achieved in a rather low number of sessions (12 or less) supporting the feasibility of these type of interventions in school environment. However, the lack of use of control groups and actual comparisons between the interventions, limit the possibility to draw firm conclusions regarding their effectiveness and thus, the results should be interpreted with caution. Confirming the effectiveness of the studied interventions requires more robust evidence and thus, improving the quality of studies in the school environment is encouraged.

## Introduction

Approximately 20% of adolescents suffer from mental disorders (1, 2). Regarding mental disorders, adolescence is a risky developmental stage; roughly 50% of all mental health disorders begin by early adolescence and 75% of them by mid-twenties (3). The most common disorders among adolescents are anxiety disorders and depression (4, 5), Mental health problems in childhood and adolescence are also strong predictors for future mental health problems (6) and elevated risk of suicide in adulthood (7). Additionally, mental health problems are associated with socioeconomic disparities related to, for example, lower education (8, 9) and unemployment (9, 10).

Taking into account the high mental and social burden associated with mental health problems and their long-term effects, it is important to offer adequate support for adolescents suffering from them. Early interventions aimed at at-risk adolescents, as well as, those suffering from mild disorders, can help to avoid transitioning to more severe mental health disorders and improve well-being (1, 11). School environment is in many ways an ideal context for promoting adolescent health, reaching mildly symptomatic adolescents, and organizing support services (12). Interventions provided in school context allows them to be reached with low threshold and minimizes the disruption to school work, which are important service features for adolescents (13). School environment poses also some challenges, for example, regarding the identification of mental health problems and adequate staffing, but in general, school-based psychotherapy interventions have shown positive outcomes (14, 15).

Different intervention methods that may be adapted for school environment are available. Cognitive-behavioral therapy (CBT) has been found to be effective in adolescents, especially in treatment of depression and anxiety disorders, and may be implemented in short therapy (16, 17). Interpersonal therapy (IPT) is based on the principle that a bidirectional link exists between interpersonal functioning and depressive symptoms; as interpersonal problems are solved, mood typically improves (18). IPT has also been developed into an application for young people (IPT-A) (19), which has been found to be effective for depression (16, 17). The evidence for CBT and IPT-A appears to be firmest in the treatment of adolescent depression, and many interventions are based on applications of the former, but also other types of interventions may be suitable and effective in treating typical adolescent mental health issues in the school context. The typical 12-session format of IPT has also been derived into a briefer application, interpersonal counseling (IPC), lasting on average 3 to 8 sessions and aimed in particular at primary care and schools to address mild to moderate depression (20). Additionally, for example, mindfulness-based interventions have been of interest (21). Taking into account a variety of potential interventions, one interesting approach is to incorporate the common elements of evidence-based interventions (22).

Compared with the intervention methods, the school environment provides even a wider variety of variables for mental health interventions, starting from the target group and setting. The provider of the intervention may be, for example, a teacher, a social worker or a health-care worker. The intervention may be targeted to, for example, healthy adolescents, at-risk adolescents or adolescents suffering from mild disorders. The targeted symptom or disorder may be depression, anxiety, substance use, eating disorders, neuropsychiatric disorders etc. The intervention may be individual or group-based. Local circumstances, for example, a high prevalence of certain problems among local adolescents, available resources and the training of staff members likely has an effect in the process of implementing a certain intervention. While the diversity likely enhances the accessibility of interventions for schools, it is a challenge regarding fidelity and hinders the effective generalization of the interventions to other contexts (23). Thus, it is important to try to identify the most effective interventions, as well as, the most effective way to deliver the interventions.

### Objective

As far as we know, no systematic reviews describing interventions delivered by health-care workers in school environments for adolescents have been published to date. Thus, our aim was to describe the evidence-based literature of these interventions for adolescents aged 12–18 with mental health problems and to assess the effectiveness of these interventions for adolescent's mental health. This review has practical importance, since it provides information to practice on what kind of mental health interventions are available and effective.

## Materials and Methods

### Eligibility Criteria

This systematic review was conducted in adherence with the PRISMA guidelines for systematic reviews to ensure a highly standardized method of the reviewing process (24). A systematic review was used in this study in order to integrate the relevant studies and provide a present scientific knowledge about the topic (25, 26). Studies were considered eligible for the review if they assessed the effectiveness of a mental health intervention in school environment, which was targeted to adolescents aged 12–18 and were delivered by a healthcare professional. The intervention had to be targeted to a mental health problem and the intervention was delivered individually to each participant. Original articles written in English were included.

### Information Sources

The original literature review was made on 15th of September in 2020. The search was conducted from the year 2000 onwards. Electronic research-literature databases searched included PubMed, CINAHL, Medline, PsycInfo, and PsycArticles. The search was limited to full-text journal articles published in peer-reviewed journals. The search strategy included terms for population, intervention, comparison and outcome, and the search strategy was modified individually to different electronic databases. Duplicates were removed and articles meeting the inclusion criteria were selected to review. Studies were discarded if the full text was not available. The detailed search strategy can be found as Supplementary Material.

### Population, Intervention, Control and Outcomes (PICO)

The following PICO criteria were used in the literature search. Population: School-aged adolescents suffering from a mental health problem. Intervention: Mental health interventions in school environments. Intervention delivered by health care professionals and delivered individually to participants. Control: Normal mental health care in community or school environment. Outcome: Reduced mental health symptoms and/or improved mental health.

### Inclusion Criteria

Studies were eligible for inclusion if the participants were 12–18-year-old adolescents with mental health problems. The interventions were delivered in a school environment by a healthcare professional. The interventions had to be targeted to mental health problems and the intervention delivered individually to each participant.

### Exclusion Criteria

Studies were excluded from the review if the intervention (1) aimed to prevent mental health problems among healthy adolescents (2) was based on a group intervention (3) was delivered by teachers, school counselors, social workers or other non-health care professionals. Studies targeted to participants with behavioral addictions, substance use, neuropsychiatric disorders and eating disorders were excluded.

Keywords relevant to adolescent population, intervention type and mental disorders were combined using standard Boolean operators. Key words were developed by consensus among the authors. One reviewer (ES) screened the titles and abstracts of the search results. The second and third reviewer (JK, OH) checked individually the consistency and accuracy of the search results.

### Selection Process and Collection Process

After the initial search was performed, the studies were screened for eligibility. The relevance of the study was assessed using first its title and abstract, and finally the full text of the paper. Full texts of potentially relevant studies were screened for inclusion individually by three authors (JK, OH, ES). Disagreements were resolved by consensus among these primary raters and a senior investigator.

### Data Extraction

The mental health outcomes of all the included studies were the main results of the review. The following information was extracted: author(s), publication year, country, population characteristics, intervention description, details from possible comparison group, outcome data on effectiveness of the intervention, and follow-up information.

### Study Risk of Bias Assessment

An assessment of the study quality was conducted. Studies were assessed independently by two authors (JK and OH) using the tool Suggested risk of bias criteria for Cochrane Effective Practice and Organisation of Care (EPOC) reviews. For unclear cases, the final decision to exclude a study was made by consensus of three authors (JK, OH, and AT). The tool is structured into a fixed set of domains of bias, focusing on different aspects of study design, conduct, and reporting (27).

### Study Selection

**Figure 1:**
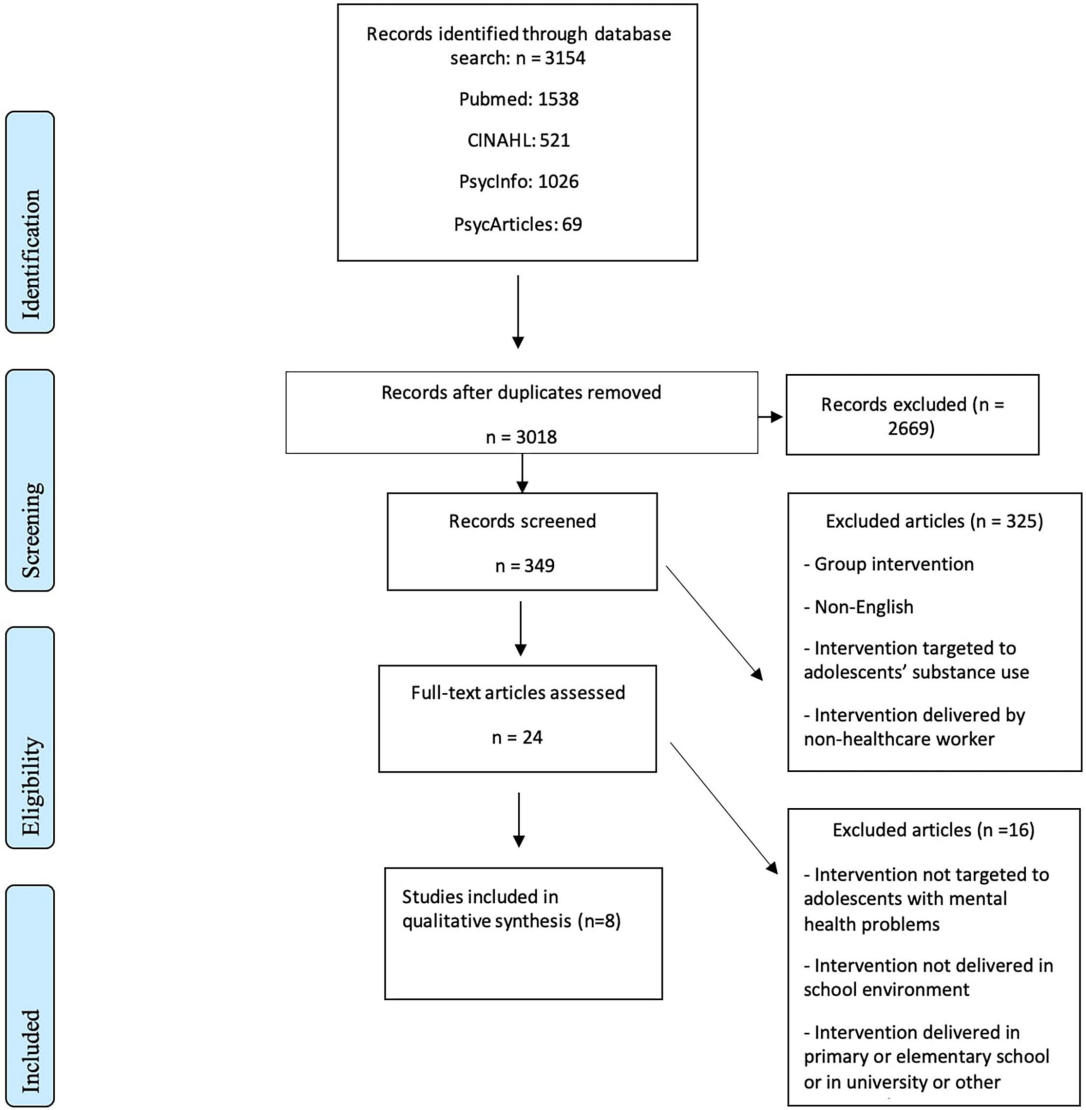
PRISMA flow chart.

Description of the results of the search and selection process from the number of records identified in the search to the number of studies included in the review are presented in PRISMA Flow chart (Figure 1).

### Synthesis Methods

Selected studies for this review were divided into two main groups based on the intervention method: (1) Cognitive-behavioral therapy interventions and (2) Other interventions. The purpose of the grouping was to determine effectiveness of the interventions and if a particular intervention is more effective than others.

## Results

### Study Characteristics

The eight studies included in the review are presented in Table 1. Five of the eight studies were conducted in the United States (28–30, 32, 33), two in Nordic countries Norway (31) and Finland (35), and one in China (34). The sample sizes ranged from 6 to 133. In all but one of the studies selected for the review, one of the main variables was depression and/or anxiety symptoms (28–32, 35).

**Table 1:** The eight studies included in the review.

**References**	**Aim(s)**	**Participants**	**Intervention description**	**Measures**	**Main outcomes**
**Cognitive-behavioral therapy interventions**					
Shirk et al. (28)	To examine predictive relations between therapeutic alliance and treatment outcomes in manual-guided, cognitive-behavioral therapy for adolescent depression.	Whole sample n = 54 36 females and 18 males.	12 manual guided sessions (50 min each) held once a week. Delivered by a psychologist	1.Computerized Diagnostic Interview scale for children (C-DISC) 2.Beck Depression Inventory (BDI) 3.Therapeutic Alliance Scale for Adolescents (TASA)	Results showed significant associations between adolescent-reported alliance and change in depressive symptoms. Both main measures for depressive symptoms (BDI, C-DISC) showed significant reductions between pre- and post-treatment assessments.
Shirk et al. (29)	To evaluate the efficacy of cognitive-behavioral therapy (CBT) for adolescent depression delivered in high schools. Outcomes were compared to results from previous efficacy trials.	Whole sample n = 50 34 females and 16 males.	12 manual guided sessions (50 min each) held once a week. Delivered by a psychologist.	1.Computerized Diagnostic Interview scale for Children (C-DISC) 2.Beck Depression Inventory (BDI) 3. Life Events Questionnaire (LEQ) 4. Trauma history (a self-report item added to the LEQ)	There was a statistically significant reduction in the depressive symptoms in the final measurements. Adolescents reporting higher stress levels, more pretreatment symptoms or a traumatic history were more likely to retain their depression after the treatment.
Michael et al. (30)	To assess the effectiveness, feasibility and acceptability of a modular common element's intervention (SEED) for treating mood disorders among middle and high school students.	Whole sample n = 20 10 females and 10 males	4–12 sessions (45 min each) held once a week across 2-3 months. Delivered by 9 clinician trainees and 1 licensed psychological associate.	1.Beck Depression Inventory (BDI-II) 2.Youth Outcome Questionnaire-30 (YOQ-30) 3.Behavioral Assessment System for Children (BASC-2)	Based on the YOQ-20 measurements, 50% of participants reported lower levels of psychological distress at post-assessment compared to their scores at baseline. Positive changes in depressive symptoms measured with the BDI were also observed
Sælid and Nordahl (31)	To examine the effects of rational emotive behaviour therapy (REBT) in reducing anxiety and depressive symptoms and increasing self-esteem and hope.	REBT n = 21 Placebo n = 21 Controls n = 20 Equal number of females and males in each group.	3 individual sessions (45 min each), approximately 2 months between sessions. Follow-up one year after the initial screening. Delivered by a certified REBT therapist.	1.Hospital Anxiety and Depression Scale (HADS) 2.Herth Hope Index (HHI) 3.Rosenberg Self-Esteem Scale (RSES) 4.Dysfunctional Attitude Scale (DAS-A)	Both REBT and ATP reduced symptoms of anxiety and depression. Both also increased self-esteem and hope, but only REBT reduced dysfunctional thinking. Overall, REBT resulted in significantly larger effects than ATP on all outcome measures, both in the short and long term.
Kirk et al. (32)	To evaluate the dose response to CBT for depressive and anxiety symptoms in a school mental health a program.	Whole sample n = 133 85 females and 48 males	An individualized treatment plan provided during school hours, with on average 14.79 sessions (range 3-55). Delivered by a psychologist and a psychologist student.	1.Youth Outcome Questionnaire (YOQ-30) 2.Behavior Assessment System for Children (BASC-2)	Participants did not improve at a steady rate. Pattern of improvement appeared to first to accelerate, then flatten and then to increase during the 14 sessions of CBT. Results indicated that, based on the YOQ-30 scores, the participants were recovered on average after 14 sessions.
**Other interventions**					
Greenwald (33)	To examine the Motivation-Adaptive Skills-Trauma Resolution (MASTR) intervention with a sample of adolescents with conduct problems.	Whole sample n = 6 1 female and 5 males	The intervention was individualized depending on the needs and circumstances. Delivered by a psychologist.	1. State-Trait Anger Expression Inventory (STAXI) 2.Children's Depression Inventory (CDI) 3.Revised Children's Manifest Anxiety Scale (RCMAS) 4.Trauma Symptom Checklist for Children (TSCC) 5.Child Report of Post-Traumatic Symptoms (CROPS) 6.Parent Report of Post-traumatic Symptoms (PROPS) 7.Problem Rating Scale (PRS)	Reductions, in particular, for post-traumatic stress and problem behaviors were observed, as well as improved school performance.
Yang et al. (34)	To examine the short- and long-term effects of attention bias modification (ABM) in adolescents with depressive disorders.	Active ABM n = 23 12 females and 11 males Placebo ABM n = 22 13 females and 9 males	8 sessions of neutral ABM over 2 weeks and 4 sessions of positive ABM over 2 weeks with a 7-week interval. Follow-up until 12 months.	1.Kiddie Schedule for Affective Disorders and Schizophrenia for School-age Children (K-SADS) 2.Center for Epidemiological Studies Depression Scale (CES-D	The attentional bias reductions were greater for the active ABM group compared with the group receiving placebo. Depressive and anxiety symptoms were significantly lower at 12-month follow up in the active ABM group.
Parhiala et al. (35)	To study the effectiveness, feasibility and acceptability of interpersonal counseling (IPC) school health and welfare services.	IPC n = 33 28 females and 5 males Brief Psychosocial Support (BPS) n = 22 15 females and 7 males	6 sessions (45 min each) of IPC or BPS over a 6 to 12-week period Delivered by school psychologists (n = 14), school social workers (n = 15) and school nurses (n = 7)	1. Beck Depression Inventory (BDI) 2. Adolescent Depression Rating Scale (ADRSc) 3. Young Person's Clinical Outcomes in Routine Evaluation (YP-CORE) 4. Children's Global Assessment Scale (C-GAS)	Based on symptom measurements, both IPC and BPS were effective in reducing depressive symptoms and improving functioning. At 6-month follow up, over 70% of adolescents were at diagnostic remission without significant group differences.

### Risk of Bias in Studies

All but two of the studies (34, 35) lacked transparency regarding how the participants were allocated. Additionally, in all but one study (34), there were problems with adequate prevention of knowledge of the allocated intervention and protection against contamination that could present a source of high or unclear risk of bias. Only three (31, 34, 35) of the eight studies included a comparison intervention or a control group. Bias assessments are reported in Table 2.

**Table 2:** Bias assessment.

	**Greenwald (33)**	**Shirk et al. (28)**	**Shirk et al. (29)**	**Michael et al. (30)**	**Yang et al. (34)**	**Sælid and Nordahl (31)**	**Kirk et al. (32)**	**Parhiala et al. (35)**
Random sequence generation	NA	NA	NA					
Allocation concealment	NA	NA	NA					
Baseline outcome measurements similar								
Baseline characteristics similar								
Incomplete outcome data								
Knowledge of the allocated interventions	NA							
adequately prevented during the study								
Protection against contamination	NA						NA	
Selective outcome reporting								

*Green, Low risk; Yellow, Unclear risk; Red, High risk; NA, Not Applicable*.

### Results of the Syntheses

#### Cognitive-Behavioral Therapy (CBT)

Although not a CBT intervention *per se*, the methods used in the common element intervention in the study by Michael et al. (30), were mostly typical elements used in CBT (e.g., psychoeducation, behavioral activation, and cognitive restructuring) and thus, the results are reported in the CBT group. In practically all of the studies in the CBT group, the intervention aimed to reduce depressive/mood symptoms.

In Shirk et al. (28) study, both post-intervention the Beck Depression Inventory (BDI) scores (*p* < 0.001), as well as total depressive symptom scores in the Computerized diagnostic interview scale for children (C-DISC) (*p* < 0.001) showed significant depressive symptom reductions. The average reduction of the BDI score was 19.87 raw points. Both adolescent and therapist reported bond (*r* = 0.76, *p* < 0.001) and collaboration (*r* = 0.73, *p* < 0.001) were significantly correlated. Adolescent reported alliance correlated significantly with change in depressive symptoms as measured both with the BDI (*r* = 0.31, *p* < 0.05) and the C-DISC (*r* = 0.37, *p* < 0.01) scores. Therapist-reported alliance was not significantly associated with change in the symptoms scores.

In Michael et al. (30) study, prior to the treatment, adolescents reported elevated levels of depressive symptoms measured with the BDI (*M* = 29.25, SD = 10.59) and distress measured with the Youth Outcomes Questionnaire (YOQ-30) (*M* = 46.35, SD = 15.59). Depressive and anxiety symptoms were also measured with the Behavior Assessment System for Children, 2nd Edition, Adolescent Form (BASC-2 SRP-A) (36). At post-test following the Student Emotional and Educational Development (SEED) intervention, participants reported on average mild to moderate levels of symptoms both for the BDI (*M* = 20.80, SD = 15.45) and the YOQ-30 (*M* = 30.10, SD=22.23) scales. Based on the BASC-SRP-A scale, altogether 60% of adolescents with at-risk or clinically significant levels of depressive symptoms and 50% of adolescents with anxiety symptoms were in the normative range at post-test.

In a randomized controlled trial (RCT) by Sælid and Nordahl (31), rational emotive behaviour therapy (REBT) was compared with attentional placebo sessions (ATP) and no sessions (control group). The change in depressive symptoms was measured with the Hospital Anxiety and Depression Scale (HADS). A significant change (*p* < 0.05) in the HADS scores between pre-test (*M* = 12.47, SD = 3.33) and one-year follow-up (post-test) (*M* = 7.21, SD = 3.53) measurements was observed for the intervention group. At follow-up, the REBT group significantly differed from the control group regarding depressive and anxiety symptoms (*p* < 0.05), but the difference between the ATP and control group was not significant. Dysfunctional thinking was significantly reduced in the REBT group from each session to session (*p* < 0.05). REBT and ATP groups also reported significant increases for hope (*p* < 0.05) and self-esteem (*p* < 0.05), although only the REBT group differed significantly from the control group.

In the study by Kirk et al. (32), participants were treated for internalizing problems, spanning both depressive and anxiety symptoms. They were provided with an individualized intervention, but virtually all participants were provided with the following core modular components of CBT: psychoeducation, self-monitoring and symptom tracking, cognitive restructuring, behavioral activation, exposure and skills training. The average number of sessions among participants was 14.79 (SD = 9.66), ranging from 3 to 55 sessions. However, only data up to session 14 ere used for analyses. The average pretreatment score on the YOQ-30 scale was 48.38 (SD = 14.99). The average amount of total reduction in the YOQ-30 score was 28.81 points (*p* < 0.001). Higher baseline score on the YOQ-30 indicated more rapid symptom improvement.

Shirk et al. (29) conducted a benchmarking study in which they compared the results of their 12-session intervention to benchmark data based on nine previous RCTs. Of the 50 participants, 39 met diagnostic criteria for major depressive disorder and 11 for dysthymic disorder. On average, the participants completed 8.8 sessions and 58% completed the full course of the intervention. A significant (*p* < 0.001) reduction of depressive symptoms based on the BDI measurements was observed. Symptom severity, life stress and trauma history were negatively related to treatment response. Compared with the benchmark studies, the initial symptom severity was relatively higher, but the intervention yielded at least the same level of results.

#### Other Interventions

##### Motivation-Adaptive Skills-Trauma Resolution (MASTR) Therapy

MASTR is a treatment method for adolescents with conduct problems (33). It includes motivational interviewing, cognitive-behavioral training and coping skills, and lastly working through traumatic material utilizing eye movement desensitization and reprocessing (EMDE). In the study by Greenwald (33), all participants made progress with some problems being resolved, while some significant problem areas remained. Actual statistical analyses were not performed due to the small number of participants and lack of control group. Five of the six families of the participants reported that they no longer needed therapy. Trauma-focused measurements showed the greatest and most consistent benefits for the participants, while modest and more inconsistent benefits were observed for depression, anxiety and anger.

##### Interpersonal Counseling (IPC) and Brief Psychological Support (BPS)

The study by Parhiala et al. (35) aimed to assess the feasibility and acceptability of IPC in school environment. The study was based on a cluster-randomization design. BPS was used as an active control intervention, and the adolescents received either IPC or BPS. IPC is a brief application of IPT and it has been developed, in particular, for schools. BPS is based on the methods and techniques used by the professionals in school health and welfare services in their routine work. Both interventions included 6 sessions and the professionals in both groups were given a one-day workshop on identification and assessment of depression. However, only the IPC counselors received method-based intervention training including 3 days of training and clinical method-specific supervision every second week for the duration of the trial. For BPS, the school workers were instructed to specifically assess and target depression symptoms thus representing “an enhanced, more intensive, and more focused version of the routine counseling (35).”

The primary outcome measures were depressive symptoms measured with the BDI and the Adolescent Depression Rating Scale clinician version (ADRSc). At post-treatment, the effect sizes of changes in the IPC group were medium (range 0.59–0.73) and for the BPS group large (range 0.83–1.53). At the end of the intervention, 48.3% of adolescents in the IPC group and 52.4% of adolescents in the BPS group achieved treatment response, which was defined as at least a 50% symptom reduction measured with the BDI scale. Similarly, 51.7% of adolescents in the IPC group and 68.2% in the BPS group achieved at least 50% symptom reduction on the ADRSc scale. No significant group differences in treatment response or recovery were detected at the end of the treatment. At 3-month follow-up, 62% of adolescents in the IPC group and 60% in the BPS (60%) were at diagnostic remission, while at 6-month follow-up, 79% of adolescents in the IPC group and 75% of adolescents in the BPS group. No significant group differences were observed in either follow-up time-point.

##### Attention Bias Modification (ABM)

Yang et al. (34) designed an ABM procedure in order to treat depressive symptoms in adolescents. Depressive and anxiety symptoms are typically associated with negative attentional biases and in ABM, dot-probe attention tasks are used to modify these. In this study, Chinese adjective word pairs were used as stimuli. As initial training, a neutral ABM procedure (90% of neutral or 10% of sad stimuli) was used followed by a positive ABM procedure (67% of positive or 33% of neutral stimuli). The placebo ABM was otherwise identical to active ABM, but shifted equally often toward neutral (50%) or sad (50%) stimuli. Both groups received 8 sessions of neutral ABM over 2 weeks, which were followed by 7-week and 9-week follow-up (pre-positive ABM) assessments. Thereafter, the groups received 4 sessions of positive ABM over 2 weeks. All 45 participants completed the neutral ABM intervention and 38 also all the positive ABM sessions.

Symptom assessments were based on several methods including, for example, the Kiddie Schedule for Affective Disorders and Schizophrenia for School-Age Children (K-SADS) (37) and the Center for Epidemiological Studies Depression Scale (CES-D). Compared with placebo, ABM showed a significant (p = 0.02) attention bias reduction for neutral ABM (p = 0.02) as well as for positive ABM (p = 0.03). Based on CES-D measurements, ABM resulted in statistically significant symptom reductions at several time-points compared with placebo. Compared with the placebo group, participants who received the ABM intervention reported lower depressive symptoms at 12-month follow-up assessment, but not at 8-month follow-up.

## Discussion

The final synthesis in the present review included 8 intervention studies published in 2002–2020. In the included studies, the intervention was delivered by a health-care worker in a school environment for adolescents aged 12–18. Majority (*n* = 5) of the interventions were based on cognitive-behavioral therapy (CBT) and in 7 studies, the main response variables include depressive symptom measurements. Overall, based on reported symptom reductions, most of the interventions ended up with good results. However, only in three studies the intervention was compared to another or the setting included a control group. Thus, actual comparisons between the effectiveness of the interventions are difficult to make. In several studies there were marked risks regarding potential bias, which also limits the possibility to draw firm conclusions.

Altogether 7 of the studies included a questionnaire-based assessment of depressive symptoms or a structured assessment for a depressive disorder. In several studies, the assessment methods were based on methods targeted to or applied for this particular age group, such as C-DISC (38) and Kiddie Schedule for Affective Disorders and Schizophrenia for School-age Children (K-SADS) (37). Given the scale of depressive symptoms affecting adolescents, it is understandable that interventions concentrating on depressive symptoms are needed. In the present review, some disorders, such as substance use disorders, were excluded in the search strategy, while the exclusion of group-based interventions likely also ruled out studies focusing on specific symptomology or disorders. For example, in particular for conduct problems, there are group-based interventions, such as Aggression Replacement Training (39), that are feasible also for school context. School environments could be useful in reaching such adolescents that may be reluctant to be remitted to mental health services. However, this review included one study concentrating on conduct problems (33). Greenwald (33) reported significant improvements in the sample, but the small sample size (*n* = 6) and high risk for bias limits the generalization of the results.

Taking into account the evidence for the effectiveness of CBT in previous literature (16, 17), not surprisingly, most studies based their interventions on its different applications. Attention bias modification (ABM) provided promising results and the study was assessed to have low risk of bias in general (34). However, the adaptation of the method to real-life school contexts remains an open question. Also the results for interpersonal counseling (IPC) were somewhat promising, but similar results were achieved with the comparison treatment (35). This is noteworthy, since only the IPC counselors received method-based intervention training and thus, the implementation required more time and financial resources. Both interventions should be further studied in other adolescent samples. There was some variability in the length of the interventions, although it is noteworthy that significant symptom reductions were achieved even in a rather low number of sessions. Kirk et al. (32) found that significant results were achieved in approximately 14 sessions, however, most of the other studies were based on a lower number of sessions. Although a challenge regarding fidelity, the low number of sessions needed to achieve significant results in school context is somewhat comforting taking into account the varying treatment adherence of adolescents, indicated, for example, by the fact that only approximately 50% of participants completed the intervention in one study (28). In the study by Sælid and Nordahl (31), good results were achieved even with only three sessions. However, it is important to note that, in their study, REBT group received homework after every session and adolescents were expected to implement what they have learned to practice. Indeed, homework is a typical feature of CBT, and may be beneficial in strengthening the effectiveness of interventions delivered typically with a low number of sessions in school context.

Most of the studies reported results based on pre- and post-intervention measurements. Only in one study based on a CBT intervention (31) and in two studies utilizing other interventions (34, 35) the authors had assessed the long-term effects of the intervention. In the study by Sælid and Nordahl (31), the follow-up assessment was conducted 1 year after the initial screening, that is, 4–6 months after the last intervention session. At follow-up, although the placebo group also differed from the controls, the rational emotive behaviour therapy (REBT) intervention group had significantly better results compared with controls. Yang et al. (34) assessed the long-term effects using several follow-up time-points. Although there was not a difference at the 8-month follow-up, at the last 12-month follow-up, those who had received the active ABM intervention reported lower depressive symptoms compared with those who had received the placebo intervention. In the study by Parhiala et al. (35), the follow-up assessments were conducted at 3-month and 6-month follow-up time-points. At 3-month follow-up, over 60% in both the IPC and control intervention (BPS) groups and at the 6-month follow-up, over 70% of participants in both groups were at diagnostic remission. These results may be evaluated as encouraging in the sense that even quite long-term effects can be achieved with different interventions, even with a small number of sessions. However, based on the results, IPC was not more effective than BPS either in the long-term and thus, it is difficult to separate to what extent recovery was associated with the specific method.

Based on current evidence, it is possible that the differences in short-term effectiveness are, regardless of the method, rather small. At least in the short-term, good results can be reached with different approaches. However, long-term follow-up needs more attention in order to clarify whether certain types of interventions provide more long-lasting results. Another key point is the cost-effectiveness of the interventions. Although this was outside the scope of this review, the descriptions of training processes were typically quite concise and superficial and thus, reliable assessments on the resource demands for the implementation of the interventions would have been difficult to make. In practice, the cost for the training and implementation of the intervention can be of major significance regarding its feasibility. It is also important to acknowledge the prerequisites and limitations that school environments pose for interventions. School professionals have to possess sufficient knowledge of mental disorders and skills to identify of them. For example, students with externalizing symptoms, who are migrants or represent minorities are at risk to be misinterpreted or remain undetected (40). Furthermore, identification has to be tied to working referral practices. The occupations and training of the staff also influence both the need for interventions and their implementation.

We used a standardized approach for the risk of bias assessment and identified several factors that indicated at least some risks for most of the studies. For example, most of the studies included in the analysis did not have any control group. Although significant symptom reductions were observed between the pre- and post-intervention assessments in the studies, this undermines certainty that these were actual treatment effects. Comparing the more recent studies with older ones, there was less potential bias related to, for example, allocation concealment and outcome reporting improving study quality. However, potential bias risks related to the prevention of knowledge of the intervention during the study and protection against contamination were low only in one study (34). Taking into account the study settings and the number of potential risks related to most of the studies, the quality of the studies also limits the possibility to draw firm conclusions.

### Limitations

The central limitations are the inclusion and exclusion criteria for this study, and the quality of the studies included in the review. The number of studies included in the final analysis was rather small. This was partly a result of the rather tight inclusion and exclusion criteria. Taking into account the wide variety of variables in school-based intervention settings (e.g., deliverer of the intervention, individual/group), the criteria were a conscious decision as an attempt to end up in a selection of at least to some extent comparable studies. The quality of the studies included in the review is also a limitation. For the most part, this limitation relates to the lack of control groups and the number of other potential biases in the studies. Although the findings in the studies indicate clear potential in reducing mental health symptoms, these issues hinder the possibility of drawing firm conclusions regarding their effectiveness.

## Conclusion

Most of the interventions included in this review provided good results, at least in the short-term, supporting their use in school environments. Based on symptom reductions, good results were reached with different kinds of methods, although the number of sessions was similar between the studies. However, due to the lack of use of control groups, short follow-up periods, and marked potential bias in several studies, the results should be interpreted with caution. Thus, improving the overall quality of future intervention studies in the school environment is encouraged in order to confirm their effectiveness.

## Data Availability Statement

The original contributions presented in the study are included in the article/Supplementary Material, further inquiries can be directed to the corresponding author.

## Author Contributions

JK and ES: formal analysis and writing—original draft preparation (equal lead). OH and AT: formal analysis, writing—review, and editing. SS: conceptualization, writing—review, and editing. MA and KK: writing—review and editing. MK: conceptualization, writing—review and editing (lead), and project administration. All authors contributed to the article and approved the submitted version.

## Funding

This research was supported by State Research Funding awarded by the Satakunta Hospital District and Turku University Hospital and grants awarded by the City of Turku/Welfare division and the Outpatient Care Research Foundation.

## Conflict of Interest

The authors declare that the research was conducted in the absence of any commercial or financial relationships that could be construed as a potential conflict of interest.

## Publisher's Note

All claims expressed in this article are solely those of the authors and do not necessarily represent those of their affiliated organizations, or those of the publisher, the editors and the reviewers. Any product that may be evaluated in this article, or claim that may be made by its manufacturer, is not guaranteed or endorsed by the publisher.
