# Supplementary material for: The Effectiveness of Individual Mental Health Interventions for Depressive, Anxiety and Conduct Disorder Symptoms in School Environment for Adolescents Aged 12–18—A Systematic Review
Source: Front Psychiatry. 2021 Dec 9;12:779933. doi: 10.3389/fpsyt.2021.779933 (PMC8695927; doi:10.3389/fpsyt.2021.779933)
Supplement: Supplementary file 1 [file Table_1.DOCX]

Supplementary table. Literature search strategy.

| **Search terms** | **Items found** |
| --- | --- |
| **1. PubMed** ("Adolescent"[Mesh] OR Adolesc*[tiab] OR Teen*[tiab] OR Yout*[tiab] OR Young*[tiab] OR Minor*[tiab] OR School-ag*[tiab] OR Child*[tiab] OR Studen*[tiab] OR freshma*[tiab] OR sophomore*[tiab] OR junio*[tiab]) AND ("Cognitive Behavioral Therapy"[Mesh] OR "Interpersonal counseli*"[tiab] OR IPC[tiab] OR "cognitive t*"[tiab] OR psychoeducat*[tiab] OR CBT[tiab] OR "cognitive-behavioral thera*"[tiab] OR "short intervent*"[tiab] OR "behavioral interventi*"[tiab] OR "Family Interventio*"[tiab] OR "Parenting intervent*"[tiab] OR "Systemic Multimodal*"[tiab]) AND ("Mental Disorders"[Mesh] OR Depress*[tiab] OR Anxie*[tiab] OR "Psychiatric illne*"[tiab] OR "Psychiatric disord*"[tiab] OR "psychiatric symptom*"[tiab] OR "Mental disord*"[tiab] OR "generalized anxiety disord*"[tiab] OR OCD[tiab] OR "obsessive compulsive disord*"[tiab] OR "panic attac*"[tiab] OR "panic disord*"[tiab] OR suicidal*[tiab] OR self-harm*[tiab] OR self-injur*[tiab] OR "behavioral disturban*"[tiab] OR "behavioral disord*"[tiab] OR "behavioral probl*"[tiab] OR "mood disord*"[tiab] OR "delusional disord*"[tiab] OR "dissociative disord*"[tiab] OR psychosi*[tiab] OR psychoti*[tiab] OR sleep*[tiab] OR "Substance-Related Disorders"[Mesh]) AND ("Schools"[Mesh] OR "School Health Services"[Mesh]OR "School Mental Health Services"[Mesh] OR schoo*[tiab]) | 1538 |
| **2.CINAHL** (MH "Adolescence+" OR Adolesc* OR Teen* OR Yout* OR Young* OR Minor* OR School-ag* OR Child* OR Studen* OR freshma* OR sophomore* OR junio*)  AND (MH "Cognitive Therapy+" OR MH "Behavior Therapy+" OR "Interpersonal counseli*" OR IPC OR "cognitive t*" OR psychoeducat* OR CBT OR CBT OR "cognitive-behavioral thera*" OR "short intervent*" OR "behavioral interventi*" OR "Family Interventio*" OR "Parenting intervent*" OR "Systemic Multimodal*") AND (MH "Substance Use Disorders+" OR MH "Behavioral and Mental Disorders+" OR "mental health*" OR Depress* OR Anxie* OR "Psychiatric illne*" OR "Psychiatric disord*" OR "psychiatric symptom*" OR "Mental disord*" OR "generalized anxiety disord*" OR OCD OR "obsessive compulsive disord*" OR "panic attac*" OR "panic disord*" OR suicidal* OR self-harm* OR self-injur* OR "behavioral disturban*" OR "behavioral disord*" OR "behavioral probl*" OR "mood disord*" OR "delusional disord*" OR "dissociative disord*" OR psychosi* OR psychoti* OR sleep*)  AND MH ("Schools+" OR schoo* OR MH "School Mental Health Services") | 521 |
| **3.APA PsycInfo and 4. APA PsycArticles** (Adolesc* OR Teen* OR Yout* OR Young* OR Minor* OR School-ag* OR Child* OR Studen* OR freshma* OR sophomore* OR junio*) AND (DE "Cognitive Therapy" OR DE "Cognitive Behavior Therapy" OR "Interpersonal counseli*" OR IPC OR "cognitive t*" OR psychoeducat* OR CBT OR "cognitive-behavioral thera*" OR "short intervent*" OR "behavioral interventi*" OR "Family Interventio*" OR "Parenting intervent*" OR "Systemic Multimodal*")  AND (DE "Mental Health" OR DE "Mental Disorders" OR "mental health*" OR Depress* OR Anxie* OR "Psychiatric illne*" OR "Psychiatric disord*" OR "psychiatric symptom*" OR "Mental disord*" OR "generalized anxiety disord*" OR OCD OR "obsessive compulsive disord*" OR "panic attac*" OR "panic disord*" OR suicidal* OR self-harm* OR self-injur* OR "behavioral disturban*" OR "behavioral disord*" OR "behavioral probl*" OR "mood disord*" OR "delusional disord*" OR "dissociative disord*" OR psychosi* OR psychoti* OR sleep*)  AND ("Schools+" OR schoo* OR DE "School Environment") | 3.1026  4.69 |
